# Paving the way in implementation of SCID newborn screening in developing nations: feasibility study and strategies to move forward in Malaysia

**DOI:** 10.3389/fimmu.2024.1400247

**Published:** 2024-06-25

**Authors:** Gaayathri Kumarasamy, Khayrin Khairiz, Wai Leng Chang, Thin Thin Aye, Adli Ali

**Affiliations:** ^1^ Arcadia Life Sciences, Hive 5, Taman Teknologi Malaysian Research Accelerator for Technology & Innovation (MRANTI), Bukit Jalil, Kuala Lumpur, Malaysia; ^2^ Department of Pediatric, Faculty of Medicine, Universiti Kebangsaan Malaysia, Kuala Lumpur, Malaysia; ^3^ Research Center, Hospital Tunku Ampuan Besar Tuanku Aishah Rohani, Universiti Kebangsaan Malaysia (UKM) Specialist Children’s Hospital, Kuala Lumpur, Malaysia; ^4^ Institute of IR4.0, Universiti Kebangsaan Malaysia, Bangi, Malaysia; ^5^ Infection and Immunology Health and Advanced Medicine Cluster, Universiti Kebangsaan Malaysia, Kuala Lumpur, Malaysia

**Keywords:** neonatal, PID, SCID, screening, implementation

## Abstract

Early diagnosis and effective management of Primary immunodeficiency diseases (PIDs), particularly severe combined immunodeficiency (SCID), play a crucial role in minimizing associated morbidities and mortality. Newborn screening (NBS) serves as a valuable tool in facilitating these efforts. Timely detection and diagnosis are essential for swiftly implementing isolation measures and ensuring prompt referral for definitive treatment, such as allogeneic hematopoietic stem cell transplantation. The utilization of comprehensive protocols and screening assays, including T cell receptor excision circles (TREC) and kappa-deleting recombination excision circles (KREC), is essential in facilitating early diagnosis of SCID and other PIDs, but their successful application requires clinical expertise and proper implementation strategy. Unfortunately, a notable challenge arises from insufficient funding for the treatment of PIDs. To address these issues, a collaborative approach is imperative, involving advancements in technology, a well-functioning healthcare system, and active engagement from stakeholders. The integration of these elements is essential for overcoming the existing challenges in NBS for PIDs. By fostering synergy between technology providers, healthcare professionals, and governmental stakeholders, we can enhance the efficiency and effectiveness of early diagnosis and intervention, ultimately improving outcomes for individuals with PIDs.

## 1 Introduction

Primary immunodeficiency diseases (PID) encompass a diverse range of immunity-related genetic abnormalities, with most cases manifesting in infancy and leading to considerable morbidity and mortality (1). To date, the extensive clinical range of PID has been acknowledged, with documentation of over 485 distinct genetic mutations leading to PID (2). The increasingly broadened spectrum of PID manifestations necessitates constant improvement in knowledge and awareness among healthcare professionals (3). The frequency of PID in Malaysia is unknown due to the absence of a national PID registry. Although a Malaysian systematic review of literature estimated the prevalence of PID to be 0.37 per 100,000 Malaysian population, this may not reflect the actual rate due to underdiagnosis and underreporting of cases. The rate of consanguinity in Malaysia is 5–9%. Notably, consanguineous marriage is not a common practice among Malaysians compared to other Southeast Asia and Middle East countries. It is practiced among indigenous populations and some ethnic groups (4). However, geographical limitations that restrict the expansion of the genetic pool, contribute to a higher incidence of genetic mutations in Malaysia. This highlights the complex interplay between genetic predisposition, environmental factors, and local variation in the prevalence of this disease.

In Malaysia, the management and research of PIDs confront numerous challenges, creating a complex landscape for healthcare professionals. Significant gaps persist in understanding PIDs, and the translation of diagnosis and management strategies into clinical practice remains a critical issue. The limited availability of comprehensive epidemiological data on PIDs, coupled with a lack of patient awareness and diagnostic facilities, poses a substantial hurdle for the healthcare community. Additionally, the field of clinical immunology is considered novel and it is not recognized as a subspecialty in Malaysia yet, contributing to insufficient immunologists in practice (5, 6). Given there is a lack of immunologists in providing the standard of care for PID patients, they face the risk of being misdiagnosed or undiagnosed (7). This challenging scenario contributes to unnecessary suffering and preventable deaths among individuals with PIDs in the country.

The limitation of data registry regarding the frequency of PIDs in Malaysia poses a significant obstacle to assess the cost-effectiveness of introducing routine screening measures. Obtaining reliable prevalence for PIDs is crucial not only for understanding the scope of the issue but also for formulating effective public health strategies. Addressing these challenges requires concerted efforts to enhance knowledge, promote awareness, and establish robust diagnostic capabilities within the healthcare system, ultimately working towards improving the overall management and outcomes for individuals with PIDs in Malaysia.

Severe combined immunodeficiency (SCID) encompasses a diverse range of genetic mutations resulting in a halt in the maturation of T lymphocytes (8). Depending on the molecular defect, B and NK cells may be variably absent in SCID, constituting an immunological emergency requiring prompt diagnosis and management, as SCID is fatal within the first year of life without treatment (1, 9).The first case of SCID in Malaysia was identified in 1993 (10). The incidence of SCID in Malaysia has been on the rise with a notable increase observed since 2010, whereby there were at least one to two cases diagnosed annually, in contrast to the six cases reported over an 18-year span from 1992 to 2010 (11). Since 2012, there has been a rising trend in this number, with an average of three to five cases of SCID annually being referred to clinical immunologists in the country. Despite this, SCID patients often face delayed diagnosis, contributing to a low survival rate, as primary physicians, including those who serve at referral centers, frequently fail to suspect it (12).

Up until 2016, the unfortunate reality was that nearly all SCID cases in Malaysia witnessed a tragic demise between four to 23 months of age. The primary cause of death was infection-induced end-organ damage, particularly affecting the lungs and liver. Although the first haploidentical hematopoietic stem cell transplantation (HSCT) was successfully conducted for SCID in Malaysia back in 2014, the transplant was only conducted when the patient was 13 months old due to lack of transplanter expertise at that time. Despite the procedure, due to pre-transplant existing chronic infection, the patient eventually succumbed before her second birthday. Various other factors contributed to this grim scenario, including the absence of a recognized family history, the lack of distinctive physical or classical characteristics with their inherent variability, limited awareness among pediatricians regarding early suspicion, and a deficiency in available facilities for conducting necessary diagnostic work-up (12).

## 2 The history of SCID newborn screening implementation - insights from T cell receptor excision circles initiatives

The initiative to screen newborns for SCID stems from previous data revealing a higher survival rate among infants who remained infection-free and underwent HSCT before the age of 3.5 months, compared to those who were transplanted when they were older (13, 14). This underscores the importance of prompt treatment following early diagnosis, which can be achieved through a newborn screening program (15). In 2005, Chan and Puck proposed the utilization of TREC assay to screen newborns for SCID and T cell lymphopenia (16). Following that, the first statewide newborn screening program for SCID was commenced in Wisconsin in 2008 (17). Subsequently, the US Secretary’s Advisory Committee on Heritable Disorders in Newborns and Children recommended the addition of SCID to the Recommended Uniform Newborn Screening Panel in 2010 (18). This screening program was later incorporated state-by-state after obtaining approval and funding from the respective state governments, eventually leading to nationwide implementation in the United States (9).

An increasing number of countries worldwide have adopted this newborn screening program, including Canada, Iceland, Ireland, Switzerland, Germany, Czech Republic, Netherlands, Denmark, Finland, Sweden, Norway, Ukraine, Israel, Qatar, New Zealand, Hong Kong, Taiwan and Singapore (1, 19–22). Additionally, some other countries have also embarked on initiatives towards SCID newborn screening program, which includes either regional implementation or a pilot project, in the United Kingdom, Italy, France, Argentina, Belgium, Spain, Slovenia, Slovakia, Bulgaria, Turkey, Poland, Belarus, Brazil, Kazakhstan, Russia, Australia, Japan, South Korea, China, Austria, Saudi Arabia, Iran, and Lebanon (19, 23–28). Thus, to the best of our knowledge, this makes up 42 countries that already have SCID newborn screening at their localities, whether at a national or regional level or as a pilot project.

With the commencement of SCID newborn screening in Switzerland in 2019, data from the first two years showed that the incidence of SCID was noted to be approximately 1 in 25,000 infants. This data was a significant increase from the estimation of 1 in 66,000 neonates prior to the implementation of screening (29), hence underscoring the importance of newborn screening in preventing underdiagnosis and underreporting. In Taiwan, SCID patients identified through the newborn screening program managed to undergo HSCT before infections occurred which resulted in a 100% survival rate (20). Interestingly, New Zealand had incorporated SCID into their national newborn screening program without a preceding pilot project as the stakeholders perceived the absolute importance of the program. Despite encountering several unanticipated issues during the early phase of implementation, such as the increased referral rate for diagnostic testing and challenges in diagnostic sample delivery, these issues were promptly resolved. This was possible given the regular review meetings and discussions conducted between the clinical immunologist and laboratory immunology team. In the first three years of screening, there were no cases of SCID which was not identified through the screening program in New Zealand (30). While, the pilot study in Israel was able to retrospectively identify seven SCID patients, leading to the implementation of SCID newborn screening (31).

In Southeast Asia (SEA), only Singapore has incorporated SCID newborn screening into its national newborn screening panel, while other neighboring countries including Malaysia have yet to follow suit (32). Following its nationwide implementation in 2019, 35,888 newborns were screened in the first year. Of these neonates screened, there were 13 cases of non-SCID T-cell lymphopenia (TCL) detected while none of those screened had SCID (21).

Malaysia might be considered to be in a stagnant status of ‘middle-income country’ given there have not been much advancements in the field of newborn screening for almost 20 years, despite its Gross Domestic Product (GDP) per capita being not far from that of a developed nation’s (33). One of the main hurdles in such effort is hugely contributed to the dedicated government’s funding for such a nationwide newborn screening program to be implemented.

Scrutinizing specifically among the 42 countries that have started the SCID newborn screening effort, there are currently seven developing countries that have initiated SCID newborn screening through the employment of different strategies. For instance, Lebanon became the first country in the Middle East and North Africa (MENA) region to implement the SCID newborn screening program, which was partially supported by the Ministry of Public Health (24). Additionally, academic research projects through two universities and the Jeffrey Modell Foundation Diagnostic and Research Center of São Paulo have led to the implementation of two pilot programs for SCID newborn screening in Brazil (25). While a pilot project in Turkey was conducted following a close collaboration between Ankara University School of Medicine and The Ministry of Health (34).

## 3 Advancement in newborn screening in PID - TREC/KREC

TRECs and KRECs are enduring DNA episomes generated during the rearrangement of T-cell and B-cell receptors in the thymus and bone marrow, which are used in newborn screening for PID. Exclusive to thymus-derived T cells, TRECs are employed in newborn screening for identifying severe T-cell lymphopenia and serve as an indicator of thymic output. TREC and KREC copy numbers can be simultaneously measured using a developed multiplex quantitative real-time polymerase chain reaction (PCR) method to identify T and B cell lymphopenias (35). The characteristics of TREC, such as its high stability, resistance to degradation, and non-replication during cell division, contribute to its advantage as a valuable indicator of thymic output (36). Besides, developing TREC assay using dried blood spots has allowed for neonatal screening of SCID as a proactive public health initiative (37).

Apart from TREC analysis, alternative methods for SCID screening have been suggested. In Italy, newborns undergo screening for SCID using the TREC assay, combined with tandem mass spectrometry for adenosine deaminase (ADA) deficiency detection. This approach also facilitates the identification of infants with delayed-onset ADA deficiency (38–40). In addition, delayed-onset ADA SCID can be detected through a biochemical assay measuring adenosine and 2’-deoxyadenosine, while those with RAG hypomorphic variants may exhibit reduced or missing B-cell kappa chain receptor excision circles (KRECs) (41).

Building on these advancements, expanded panels now leverage state-of-the-art technologies such as Tandem Mass Spectrometry (MS/MS). This enables the detection of various metabolic disorders, including amino acid disorders, organic acidemias, and fatty acid oxidation disorders, with high sensitivity and specificity. Moreover, MS/MS has introduced the idea of conducting multiple metabolite analyses to identify numerous metabolic disorders in a single analytical run (42). Another powerful tool, Next-Generation Sequencing (NGS), can identify genetic mutations associated with a wide range of rare diseases (43). NGS allows for the rapid sequencing of an individual’s entire genome or targeted gene panels, further enhancing the capability of newborn screening programs (44, 45).

Multiplex assays play a vital role in newborn screening programs by enabling the simultaneous and comprehensive analysis of a wide range of metabolic disorders, genetic conditions, and other health markers in newborns (46). These assays leverage techniques such as multiplexed PCR (47, 48) to detect and quantify rare diseases such as SCID and spinal muscular atrophy (SMA), enhancing the efficiency and accuracy of newborn screening. Furthermore, advancements in automation and robotics have given rise to high-throughput screening platforms that can rapidly process large numbers of samples. These platforms streamline the screening process, boosting throughput, reducing turnaround times, and facilitating early detection and intervention.

AI/ML-based NBS offers new possibilities for reducing false positive rates and uncovering previously unknown disease patterns by leveraging complex combinations of features instead of predefined cut-off values (49). These mathematical approaches are complementary to traditional biochemical and genetic tests, aiming to enhance the diagnostic specificity of NBS programs through second and multiple-tier analyses.

Overall, these technological advancements in combination with AI/ML have played a role in shaping the progress of newborn screening for rare diseases, allowing for earlier detection, providing more comprehensive diagnostic information, and ultimately leading to improved outcomes for individuals and their families affected by these conditions. Ongoing research and innovation in this field are crucial to continually enhance the effectiveness and accessibility of newborn screening programs on a global scale.

## 4 Economic implication for extended newborn screening

Malaysia, with a total population of 32.7 million, has approximately 28% children under the age of 18. In terms of newborns, there were 423,124 live births recorded in 2022, with an estimated life expectancy of 73.4 years. The neonatal mortality rate stands at 4.2 per 1,000 live births (equivalent to 2000 newborns) (50). If every newborn is screened, the Total Available Market (TAM) is estimated to be around MYR 1.7 billion. With private hospitals accounting for 60% and public hospitals for 40% of the market, the estimated Serviceable Addressable Market (SAM) for Expanded Newborn Screening (ENBS) is approximately MYR 1.2 billion and MYR 680 million respectively. However, this rough estimation is based on the pricing of commercially available ENBS, such as Inborn Errors of Metabolism (IEM) screening which is often packaged with maternity tests. Nevertheless, it provides a basic understanding of the potential market size of ENBS screening in Malaysia. In the United States, the cost of SCID screenings ranges from USD 4–6 (51, 52), highlighting a potential pricing reference for future implementation in Malaysia (45).

According to a study done in Ireland, the noenatal mortality rate for rare diseases is at 55.6% and this is projected to impact 1000 Malaysian newborns in a year (53). Specifically, the incidence rate for SCID is estimated to be 1 in 58,000 newborns (54), resulting in an estimation of eight cases annually in Malaysia. Among various rare diseases, SCID is a treatable disease through HSCT. However, the effectiveness of treatment decreases with delayed detection (13, 55). Late diagnosis and treatment of SCID can lead to developmental disabilities, an increased risk of multiple infections, and ultimately, mortality (56). On top of the clinical aspect, financially, one of the hospitals in the United States reported that the total charge for the early diagnosed case was approximately USD 607,000 versus a median charge of USD 1.9M for the late diagnosed cases (57).

In Malaysia, the treatment cost for SCID is estimated between MYR 50,000 to half a million depending on the hospitals. Treatment alone amounted to a minimum of MYR 1 million per individual case, excluding health system costs (i.e. primary care, specialist treatment and follow-ups, pharmaceuticals, transportation, etc) hence late detection will cause treatment repetition or prolonged hospitalizations adding to the costs (12, 58). It was reported that children who receive immunoglobulin replacement therapy (IGRT) recorded low school functioning compared to those without IGRT. This is primarily due to the need for frequent hospital visits and other health-related issues, which adversely affect the overall quality of life for these children (59). It is proven that utilizing NBS/ENBS for SCID screening leads to significant benefits. In Australia, early HSCT resulted in savings of USD 123,000 and an additional 1.53 Quality-adjusted life years (QALYs) per child diagnosed with SCID over a 5-year period (60).

Economically with the adaptation of new screening methods such as multiplex qPCRs, MS/MS, AI/ML, and NGS, it is expected to influence firstly the growth of both clinical and technical expertise. This is due to the increase of skilled workforce to aid in the management of the introduction of new specializations like genetic counselors. Secondly, localization of commercially available ENBS testing through technological service providers in the country opens up technological accessibility as well as job growth for either value-added skills to the current workforce or new recruitments within commercial laboratories. Thirdly, we can expect a spillover to other industries such as pharmaceuticals and medical tourism (61) once Malaysia has managed to formalize the algorithm for disease management and treatment.

Overall, while there are upfront costs associated with implementing newborn screening for SCID, the potential long-term economic benefits, including healthcare savings and improved quality of life for affected individuals, suggest a favorable economic outlook for such programs.

## 5 Pathway for enabling implementation of ENBS

In Malaysia, healthcare services are provided by both the public and private sectors. The Ministry of Health (MoH) oversees the majority of healthcare services in the public sector, which are tax-funded and heavily subsidized, with patients being charged a nominal fee for the services provided. In the private sector, healthcare services are primarily accessed through fee-for-service arrangements, with patients either paying out of pocket (OOP) or covered by private health insurance (PHI) (62, 63). Hence, the nationwide implementation of ENBS presents a potential challenge as it may strain the capacities of the public healthcare system. To address this challenge, a preliminary strategic approach should involve government collaboration with the private sector, including private health insurance (PHI) providers, healthcare providers, and non-governmental organizations (NGOs), forming a public-private partnership (PPP).

## 6 Developing national guidelines

The protocols for newborn screening in Malaysia typically include screening for congenital disorders such as congenital hypothyroidism, and glucose-6-phosphate dehydrogenase (G6PD) deficiency for all newborns. Hence, collaboration between healthcare professionals, experts, and relevant stakeholders is needed to ensure that newborn screening practices are aligned with international standards and best practices. It’s important to note that guidelines and protocols for newborn screening may evolve over time based on scientific advancements, changes in disease prevalence, and other factors.

## 7 Sustainability and accessibility of new ENBS technology and infrastructure

Research and development (R&D) is essential for advancing and expanding NBS programs. It involves identifying new biomarkers, innovating screening technologies, developing multiplex assays, advancing genomic sequencing, improving data analysis tools, standardizing screening procedures, and evaluating health outcomes. The major challenge in rare disease management is financial limitations. Due to the rare nature of the diseases and lack of attention from funders, only a minimal amount of funds is allocated for managing rare diseases. Moreover, in Malaysia, R&D is primarily driven by universities where universities seek grants to initiate pilot projects and acquire new technology, addressing some technical expertise and infrastructure limitations. However, the outcomes from these academic studies are often published but not integrated into MoH protocols (64). Therefore, relying solely on grant funding poses risks, such as funds depletion or project termination due to lack of progress for further advancement or product. Thus, depending solely on grants is unsustainable for long-term projects such as ENBS. To achieve sustainable funding and effective technology advancement, universities could explore establishing spin-off companies such as Plutonet and Zakesy Biotech (65, 66) to formalize research groups as separate entities, attracting investors from public and private funding sources.

NBS in Malaysia currently covered by the government’s tax-funded healthcare system includes G6PD deficiency, Congenital Hypothyroidism, hearing test, and critical congenital heart disease for infants with symptoms. The cost and payment for other NBS screening (IEM and IMDs) are partially covered by the government’s tax-funded healthcare system, allowing the public to access services through both public and private healthcare providers. However, screening methods for rare diseases such as PID (i.e., TREC and KREC) are not readily available at public facilities, necessitating referral to private providers for ENBS testing. For individuals choosing private healthcare, NBS screening is typically included in insurance maternity packages, with costs ranging from MYR 3,700 to MYR 5,500. This suggests the potential for ENBS testing inclusion. Nonetheless, it remains unclear whether ENBS testing is conducted in-house or outsourced to reference laboratories like the Ministry of Health’s Specialized Diagnostics Centre (SDC), as this information is not highlighted in promotional materials (67, 68).

## 8 Integrating screening into healthcare system

Another strategy could be the implementation of ENBS screening regionally, as demonstrated in Japan. For instance, the Tokyo Health Service Association offers an ENBS service in the Tokyo Metropolitan area, supplementing the free 20 conditions provided by the national program. These conditions include SCID, SMA, B Cell Deficiency (BCD), Fabry disease, Mucopolysaccharidosis Type I and Type II, and Pompe disease (69). Similarly, in Osaka, a pilot study for SMA-NBS screening has been initiated to advocate for its inclusion in Japan’s national ENBS program (70). In Malaysia, state-level health initiative programs such as SELHEP (Selangor Health Partnership) could serve as platforms for introducing ENBS at the state level before scaling it nationally. SELHEP focuses on priority areas such as mental health, cervical cancer, healthy aging, non-communicable diseases, and early childhood nutrition (71).

The cost of curative treatment for SCID in government hospitals ranges from approximately MYR 50,000 to MYR 100,000, while in private hospitals, it ranges from MYR 200,000 to MYR 400,000. **Table 1** shows private healthcare insurance (PHI) that offers coverage for different congenital conditions with each having a unique set of conditions and age limits for coverage. Currently, newborns are covered by PHI from 13 weeks of gestation up to 25 years of age. Coverage terms vary across policies, with most including congenital defects and child developmental disorders with a price up to MYR 30,000. However, some policies exclude coverage for functional birth defects such as neurodevelopmental, immune system, and metabolic disorders. With this set precedence, it may pose a financial challenge for newborns and children with PID if it is adopted widely by PHIs in Malaysia. Given the significant costs involved, collaborative efforts between the government and PHI providers are essential to assist parents in preparing for their newborns’ healthcare needs.

**Table 1:** Private Healthcare Insurance (PHI) currently offered for newborns and children in Malaysia.

Private Healthcare Insurance (PHI) Company, Product	Conditions Covered	Coverage Age	Policy Coverage
Company A	Congenital Conditions 1. Ventricular Septal Defect 2. Cerebral Palsy 3. Atrial Septal Defect 4. Tetralogy of Fallot 5. Transposition of Great Vessels 6. Coarctation of the Aorta 7. Infantile Hydrocephalus 8. Spina Bifida 9. Cleft Lip with/without Cleft Palate 10. Congenital Cataract 11. Congenital Deafness 12. Anal atresia 13. Esophageal Atresia 14. Congenital Diaphragmatic Hernia 15. Tracheoesophageal Fistula 16. Absence of two limbs 17. Down Syndrome 18. Truncus Arteriosis 19. Retinopathy of Prematurity	Up to 5 years old	up to MYR 30,000 per year shared between Congenital and Child Development Conditions
Company A	Child Development Disorder 1. Autism Spectrum Disorder 2. Attention Deficit Hyperactivity Disorder 3. Dyslexia 4. Gross Motor or Speech Developmental Delay	Up to 5 years old	up to MYR 30,000 per year shared between Congenital and Child Development Conditions
Company B	Congenital Conditions (27 listed congenital conditions* and ALL structural congenital conditions) We do not cover functional birth defects (other than listed Congenital Conditions) which are related to a problem with how the body system works.	Up to 7 years old	up to MYR 50,000 per year
Company B	Child Developmental Disorder (Autism, Attention Deficit Hyperactivity Disorder (ADHD), and more).	Up to 7 years old	up to MYR 8,000
Company C	Package A Severe Asthma Rheumatic Fever with Valvular Impairment Kawasaki Disease with Heart Complications Hand, Foot and Mouth Diseases with Severe Complications Severe Dengue Hemorrhagic Fever Severe Epilepsy Infantile Bacterial Meningitis Infantile Encephalitis	up to 25 years old	50% payout from the total insurance
Company C	Package B Leukemia Bone Marrow Transplant Insulin Dependent Diabetes Mellitus Intellectual Impairment Due to Illnesses or Accident Glomerulonephritis with Nephrotic Syndrome Systemic Juvenile Chronic Arthritis (Still’s Disease) Poliomyelitis Primary (Idiopathic) Cardiomyopathy Osteogenesis Imperfecta Severe Hemophilia Brain Surgery Autistic Spectrum Disorder	up to 25 years old	100% payout from the total insurance

## 9 Future outlook and conclusion

The rising incidence of PID and its associated mortality demand prompt action. The advancement of screening technologies, coupled with comprehensive training programs and collaborative efforts, offers a promising path forward. These include the enforcement of the national plan for rare diseases, establishing a national rare disease registry, and ensuring access to resources such as funding and expertise across the country. Additionally, addressing the availability and accessibility of screening methods for rare diseases in public healthcare facilities is essential. Collaborative efforts among stakeholders, including healthcare providers, governance entities, policymakers, and insurance providers, are crucial for enhancing rare disease management and NBS outcomes. It is a big feat to prioritize resources for screening programs nevertheless the examples of other nations and Malaysia itself having already taken some measures for NBS, should continue to expand on current testing modalities and with re-envisioning Malaysia NBS framework and growth of expertise, Malaysia can be brought up to speed with other nations.

## Data availability statement

The original contributions presented in the study are included in the article/supplementary material. Further inquiries can be directed to the corresponding author.

## Author contributions

GK: Writing – review & editing, Writing – original draft. KK: Writing – review & editing, Writing – original draft. WC: Writing – review & editing, Writing – original draft. TA: Supervision, Conceptualization, Writing – review & editing, Writing – original draft. AA: Supervision, Conceptualization, Writing – review & editing, Writing – original draft.
